# Effects of Serum From Radiofrequency Ablation Patients Receiving General Anesthesia or Local Anesthesia on Hepatocellular Carcinoma Cancer Cell Malignancy: A Prospective Randomized Controlled Trial

**DOI:** 10.3389/fonc.2021.686294

**Published:** 2021-09-23

**Authors:** Yumiao Shi, Tong Wu, Tao Wang, Yan Liu, Xiaoqiang Wang, Jiamei Luo, Diansan Su, Bo Zhai, Jie Tian

**Affiliations:** ^1^ Department of Anesthesiology, Renji Hospital, Shanghai Jiaotong University School of Medicine, Shanghai, China; ^2^ Department of Interventional Oncology, Renji Hospital, Shanghai Jiaotong University School of Medicine, Shanghai, China

**Keywords:** general anesthesia, local anesthesia, hepatocellular carcinoma, serum milieu, cancer cell malignancy

## Abstract

**Background:**

Whether anesthesia methods affect malignant biological behavior of cancer remains unresolved. In this study, we aim to compare the effects of general anesthesia (GA) and local anesthesia (LA) on serum collected from primary hepatocellular carcinoma (HCC) patients presenting for radiofrequency ablation (RFA).

**Methods:**

From August 2020 to December 2020, a prospective, randomized, and controlled study was conducted at Renji Hospital, which is affiliated with Shanghai Jiaotong University School of Medicine. 25 qualified patients from 18 to 65 years of age undergoing RFA were enrolled in the study and randomly assigned into two groups: the GA group (*n* = 14) and the LA group (*n* = 11). Venous blood was drawn from all patients preoperatively and 1 hour postoperatively. The serum collected was then used for the culturing of HepG2 cells. The malignant biological behaviors of HepG2 cells, including invasion, migration and proliferation, were observed after 24 hours of exposure to patients’ serum. ELISA was used to compare expression levels of pro-inflammatory cytokines (IL-1β, IL-6, TNF-α) and lymphokines (IFN-γ, IL-2) in patients’ serum from both groups.

**Results:**

HepG2 cells cultured with postoperative serum obtained from patients who received GA, but not LA, were associated with significantly increased cell invasion, migration and proliferation, compared to preoperative serum from the same patient group. Expression levels of pro-inflammatory cytokines were significantly higher, and lymphokines significantly lower in postoperative serum from GA patients compared to the corresponding preoperative serum.

**Conclusion:**

GA affects the serum milieu of patients with HCC, promoting the malignant biological behavior of a human HCC cell line.

## Introduction

Hepatocellular carcinoma (HCC), one of the most aggressive cancers worldwide (1, 2), is the second leading cause of cancer-related death in China and has a poor prognosis (3). To date, surgical resection is the main treatment for HCC. However, recurrence and metastasis rate of HCC after surgery remains high, which greatly affects patient prognosis (4). Studies have shown that recurrence and metastasis rates of large HCC after resection is greater than 60% and that of small HCC is over 40% (5). Even liver transplantation cannot completely eliminate the recurrence of HCC (6, 7). Therefore, to effectively prevent HCC recurrence and metastasis is key to improve long-term survival of HCC patients.

The perioperative time period is a dangerous window for tumor metastasis (8), among which anesthesia contributes a significant part. On one hand, the use of anesthesia reduces patients’ pain and relieves stress caused by the surgery; on the other hand, various anesthetics may affect cancer recurrence and metastasis. In recent years, a number of studies have shown that choice of anesthesia methods and drugs have a potential impact on the long-term prognosis of cancer patients (9–13). For example, Lin et al. found that patients who received general anesthesia (GA) combined with epidural anesthesia during ovarian serous adenocarcinoma surgery have a reduced mortality rate during the initial follow-up years compared to patients who received GA alone (10). However, most previous studies are comparisons between GA and GA combined with regional anesthesia, therefore it cannot be assessed whether the differences are due to a tumor-promoting effect of GA itself, or a protective effect of regional anesthesia.

Radiofrequency ablation (RFA) is a minimally invasive therapy for the treatment of HCC without damage to adjacent healthy tissue and with a shorter recovery period compared to surgical resection. It has become a safe and effective treatment for patients with HCC and is clinically widely used (14). Either GA or local anesthesia (LA) can be used to complete the operation, especially when the tumor size is still small. Our previous multi-center retrospective cohort study has shown that the anesthesia approach could influence the prognosis of HCC patients. GA patients undergoing RFA displayed a higher rate of tumor recurrence and shorter overall survival compared to HCC patients who received LA (15). However, how anesthesia methods influence the outcome of HCC patients receiving RFA surgery remains unclear.

To explore the potential mechanisms underlying the effects of anesthesia on clinical HCC patients, we performed a randomized clinical trial, allocating HCC patients to receiving either GA alone or LA alone. Serum was collected both pre- and postoperatively during the perioperative period. We then compared its effects on the malignant behaviors of HepG2 cells, a human HCC cell line, when culturing them using the collected patient serum. Our results show that for GA patients undergoing RFA, serum milieu was influenced such that GA increased the malignancy of HCC cells.

## Material and Methods

### Study Design

This was a prospective, randomized, and controlled study designed in accordance with the CONSORT recommendation.

### Patients

The study was approved by the Institutional Human Ethics Committee of Renji Hospital (2015-064), located at 160 Pujian Road, Pudong New Area, Shanghai, China, and was registered at ClinicalTrials.gov (NCT04510935). It was conducted at the Renji Hospital, Shanghai Jiao Tong University School of Medicine between August 2020 and December 2020. Informed consents were obtained from all patients or legally authorized representatives. Only patients diagnosed with HCC and undergoing elective RFA surgery were enrolled in the study. Other inclusion criteria were: (a) between 18 and 65 years of age; (b) ASA Classes I‐III; (c) the summary of the long diameter of all tumors was ≤3 cm; and (d) Child-Pugh degree A or B. Patients were excluded if they (a) had a previous elective RFA; (b) had severe systemic disease (heart, lung, kidney, or immune system); (c) INR>1.5 or platelet count <45,000 cells/mm^3^; (d) were addicted to opioids; or (e) with known extension beyond the liver.

### Randomization and Blinding

Eligible patients were randomly allocated 1:1 to receive either GA or LA according to computer-generated codes. The PROC program in SAS (version 9.0, SAS Institute Inc) was used to generate the sample randomization sequence with a 1:1 allocation. This was an open-label study, since blinding of either patients or investigators was not possible. The investigator who carried out the cell culture studies using the patients’ serum, was blinded to the treatment assignment.

### Procedures

Patients in the GA group were induced with 0.05-0.1mg/kg intravenous midazolam, 3-6μg/kg fentanyl, 1.0-2.5mg/kg propofol and 0.1-0.2mg/kg cisatracurium. A laryngeal mask was inserted for mechanical ventilation. Anesthesia was maintained with 4-8mg/kg/h propofol and 0.1-0.3μg/kg/min remifentanil, and additional non-depolarizing muscle relaxant when necessary. Patients recovered in a Post Anesthesia Care Unit (PACU), and were administered neostigmine combined with atropine routinely to reverse muscle relaxants.

Patients in the LA group were injected subcutaneously with ~10ml of 2% lidocaine at the surgical puncture points before insertion of laparoscopic needles. No propofol or other sedatives or narcotics were given. Patients were awake and breathing spontaneously during surgery.

Venous blood was obtained from patients from both groups immediately after entering the operating room and 1 hour postoperatively. Samples were centrifuged at 3000 rpm for 10 minutes at 4°C and serum was collected and stored at −80°C for future use.

### Data Collection and Outcome Measures

Personal health records of the study participants were obtained from the hospital medical record system. Primary outcome was the mean percentage change from post- to preoperative values of the invasion ability of HepG2 cells cultured with the patients’ serum for 24h. Secondary outcomes were the mean percentage change from post- to preoperative values of the migration and proliferation ability of HepG2 cells cultured with patients’ serum for 24h, and expression levels of key cytokines, including interleukin-1β (IL-1β), tumor necrosis factor α (TNF-α), interleukin-6 (IL-6), interferon gamma (IFN-γ) and interleukin-2 (IL-2), in pre- and postoperative serum.

### Cell Culture

The human HCC cell line HepG2 was purchased from the FuHeng Cell Center (Shanghai, China). HepG2 cells were cultured at 37°C in a humidified incubator containing 5% CO_2_, using high glucose Dulbecco’s modified Eagle’s medium (DMEM, Gibco, USA) supplemented with 10% fetal bovine serum (FBS, Gibco, USA) and 50 U/ml penicillin and 50µg/ml streptomycin. Cells were serum-starved in DMEM for 8-12h before treatment with DMEM plus 10% patient serum for 24h.

### Transwell Assays

Cell invasion ability was determined using Transwell chambers with an 8μm pore size (Corning, USA) and Matrigel (BD Bioscience, China). Cells were incubated in the upper chamber at a density of 2 × 10^4^ cells/chamber with 0.5mg/L Matrigel, and medium with patient serum in a final concentration of 10% was added to the lower chamber. After a 24h incubation, chambers were fixed with paraformaldehyde for 30 min and stained with 0.5% crystal violet for 20 min. Positive cells were visualized using a microscope. Three random fields per chamber were counted using the Image J1.54 software and averages were calculated to reflect invasion activity of the sample. Mean percentage change from post- to preoperative values for each individual patient was calculated and compared between the GA and LA groups. Mean percentage change = [(No. of invaded cells with postoperative serum) − (No. of invaded cells with preoperative serum)]/(No. of invaded cells with preoperative serum) ×100%. Representative fields were photographed with an Olympus fluorescence microscope at 100× magnification.

### Wound Healing Assays

Migration activity of HepG2 cells was analyzed using scratch assays. 2× 10^5^ cells per well were seeded into 12-well plates and grown to 90% confluency. 200μl pipette tips were used to draw one straight scratch per well. Cells were then washed with PBS and cultured for 24h in medium containing 10% of the patients’ serum. Three microscope images were taken of each set at 0h and 24h respectively, with the distance of cell migration measured for statistical analysis. Averages were calculated to reflect the migration activity of the sample: Recovery ratio = [(Blank area at 0h) - (Blank area at 24h)]/(Blank area at 0h) × 100%. Mean percentage change = [(Recovery ratio with postoperative serum) − (Recovery ratio with preoperative serum)]/(Recovery ratio with preoperative serum) ×100%.

### Cell Counting Kit (CCK-8) Assays

Cell proliferation ability was measured with a CCK-8 Kit. Cells were seeded in 96-well plates at a density of 6 × 10^3^ cells/well and using 10% of the patients’ serum. After 24h or 48h of culture time, 10μl CCK-8 (Yeasen Biotech Co., Ltd. Shanghai, China) was added to the cultures and cells were incubated for 30 minutes. Optical density (OD) at 450 nm was detected by a microplate reader (Berthold Technologies-TriStar2LB942, German). Each treatment was performed in six replicates. Averages were calculated to reflect proliferation activity of the sample. The mean percentage change from post- to preoperative values for each individual patient was calculated and compared between the GA and LA groups. Mean percentage change = [(OD Value with postoperative serum) − (OD Value with preoperative serum)]/(OD Value with preoperative serum) ×100%.

### EdU Assays

EdU assays were performed to investigate differences in HepG2 DNA synthesis and cell proliferation. HepG2 cells were seeded in 96‐well plates at a density of 6 × 10^3^ cells/well. EdU incorporation experiments were performed following the manufacturer’s instructions (Yeasen Biotech Co., Ltd. China). Nuclei were stained with 4′,6- diamidino-2-phenylindole (DAPI). Cells were visualized using a confocal microscope (Olympus, Japan): EdU^+^ cells (%) = number of positive EdU cells/the total number of nuclei×100%. The mean percentage change from post- to preoperative values for each individual patient was calculated and compared between the GA and LA groups. Mean percentage change = [(%EdU ^+^ cells with postoperative serum) − (%EdU ^+^ cells with preoperative serum)]/(%EdU ^+^ cells with preoperative serum) × 100%.

### ELISAs

Serum levels of pro-inflammatory cytokines and lymphokines were measured using commercial ELISA kits (R&D Systems, Inc., USA) following the manufacturer’s instructions. Catalog numbers are: interleukin-1β (IL-1β) (DLB50), tumor necrosis factorα (TNF-α) (DTA00D), interleukin-6 (IL-6) (D6050), interferon gamma (IFN-γ) (DIF50C) and interleukin-2 (IL-2) (D2050).

### Statistical Analysis

PASS (version 11.0, NCSS, LLC) software was used for sample size calculations. Since evidence on the effects of GA on the invasion ability of cancer cells was lacking, we adopted a conservative approach and assumed that the expected effect size (Cohen’s d) between groups would be small (0.3). Thus, assuming that GA would result in an 18% increase in the mean percentage change from post- to preoperative invasion, with a SD of 10%, the study would require 8 patients per group to reach 90% power with an α equal to 0.05. When including an attrition rate of 10%, 9 patients per group should be included.

SPSS (version 24.0, SPSS Inc, Chicago, USA) software and GraphPad Prism 8 (GraphPad Software, San Diego, CA) were used for data analysis. Continuous variables were expressed as mean ± standard deviation (Mean ± SD) after they were proven to be normally distributed using the Kolmogorov-Smirnov (K-S) test. When comparing cell invasion, proliferation and migration ability in pre- or postoperative serum from the two groups, multiple comparisons were performed using two‐way ANOVA. Mean percentage changes from post- to preoperative values between the two groups were calculated using Student’s *t*-tests. Categorical variables were compared using the χ2 test with the Yates correction or Fisher’s exact test (when total sample size was <40 or the expected frequency was <1). Two‐sided tests were used and *P*-values <0.05 were considered significant.

## Results

**Figure 1:**
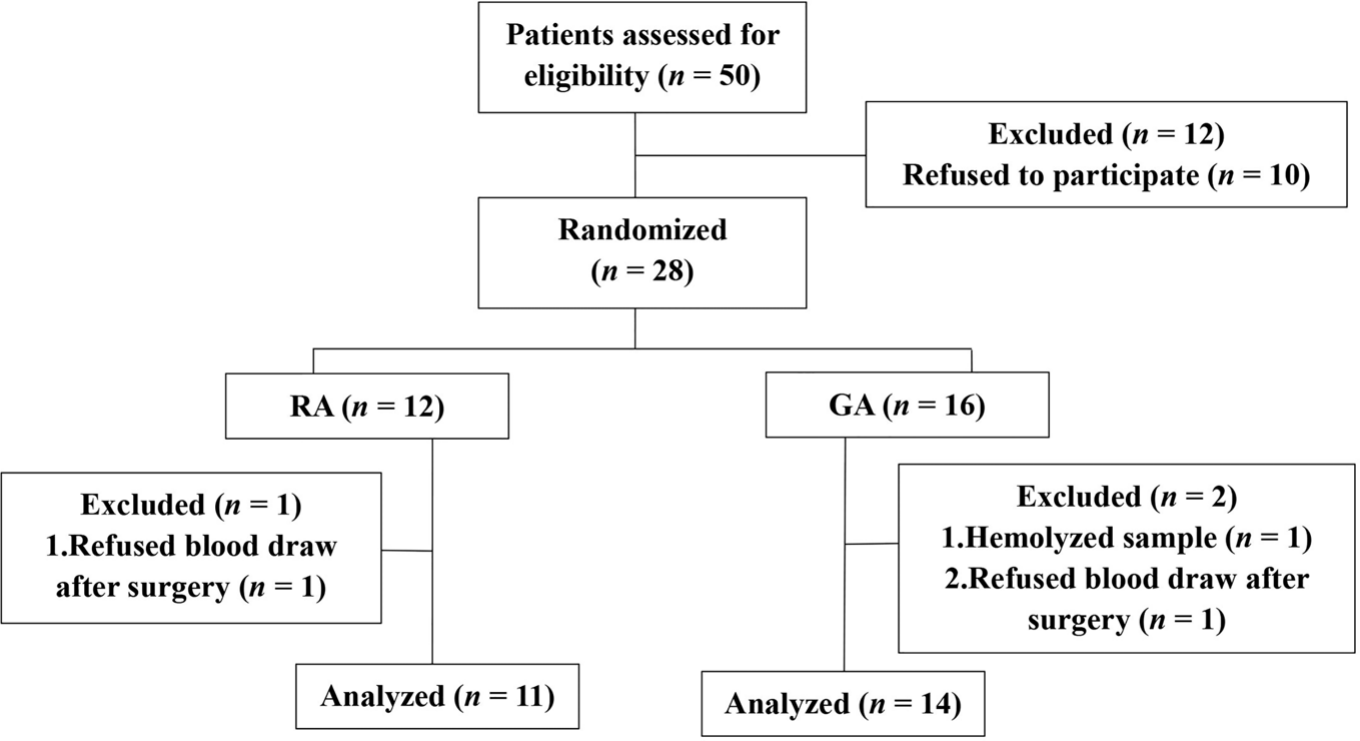
Clinical trial flow diagram. 28 patients were recruited and randomized to the LA group or GA group. 25 patients were included in the final analysis, *n* = 11, LA group, and *n* = 14, GA group.

From August 2020 to December 2020, a total of 28 patients were recruited and randomized into the LA or GA groups. Among these, two refused the postsurgical blood draw and one sample hemolyzed. Therefore, 25 patients were included in the final analysis (**Figure 1**). More than ten variables were analyzed and compared between the two groups, including general patient information, operation time, liver function variables and cancer characteristics, and none of the differences reached statistical significance (**Table 1**).

**Table 1:** General characteristics of the thermal ablation surgery patients [Mean (SD) or number].

Variables	LA (*n* = 11)	GA (*n* = 14)	*P* value
Sex			
Male	9	11	1
Female	2	3	
Age (y)	54.8 (11.62)	52.0 (10.64)	0.54
Height (cm)	170.4 (7.10)	170.7 (7.20)	0.92
Weight (kg)	67.3 (11.95)	69.5 (16.53)	0.71
ASA (I/II/III)	0/11/0	0/14/0	1
Hypertension (yes/no)	1/10	2/12	1
Diabetes (yes/no)	1/10	1/13	1
Cirrhocsis (yes/no)	4/7	2/12	0.35
HBV/HCV infection (yes/no)	6/5	8/6	1
Child-pugh stage (A/B)	11/0	14/0	1
Adjuvant chemoradiotherapy* yes/no)	5/7	6/8	1
Tumor size (cm)	1.9 (0.54)	1.9 (0.62)	0.89
ALB (g/L)	44.3 (3.23)	42.4 (4.70)	0.26
ALT (U/L)	28.1 (14.88)	26.1 (16.03)	0.75
AST (U/L)	23.5 (5.25)	25.7 (13.10)	0.61
TBIL (mmol/L)	12.4 (6.44)	12.1 (6.50)	0.91
Duration of surgery(min)	12.4 (5.15)	12.6 (4.52)	0.99

Variables are shown as “mean (SD)”. *Adjuvant chemoradiotherapy is defined as patients received transcatheter arterial chemoembolization (TACE) or radioactive seed implantation simultaneously with or after TA surgery. ASA, American Society of Anesthesiologists; HBV, hepatitis B virus; HCV, hepatitis C virus; ALB, serum albumin; ALT, Alanine transaminase; AST, aspartate aminotransferase; TBIL, total bilirubin; SD, standard deviation; LA, local anesthesia; GA, general anesthesia.

### Serum From RFA Patients Receiving GA Facilitated Cell Invasion in HepG2 Cells

**Figure 2:**
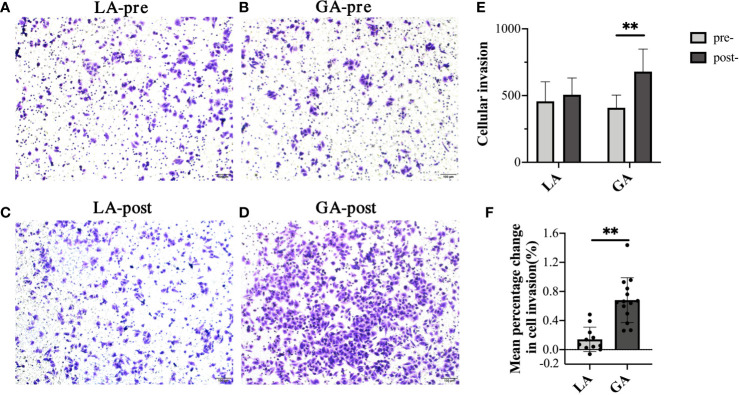
Serum from RFA patients receiving GA facilitated cell invasion of HepG2 cells. **(A)** Representative image of pre-LA serum-treated HepG2 cells; **(B)** Representative image of pre-GA serum-treated HepG2 cells; **(C)** Representative image of post-LA serum-treated HepG2 cells; **(D)** Representative image of post-GA serum-treated HepG2 cells; Original magnification, 100X; **(E)** Graphical representation of cells that invaded the lower surface of the four groups; **(F)** Graphical representation of the mean percentage change from post- to preoperative values of invading cell numbers in the GA group *vs* the LA group. Values are expressed as mean ± SD. *n* = 11, LA group. *n* = 14, GA group. **P < 0.01. GA, General Anesthesia; LA, Local Anesthesia.

Transwell assays were used to investigate the invasion ability of HepG2 cells. As shown in **Figures 2A–E**, there was no significant difference in invasion ability of cells when they were treated with preoperative (pre-) serum from the GA or LA groups. Interestingly, it clearly showed that the number of cells invading to the lower surface was significantly greater when treated with serum from the postoperative (post-) GA group compared to the pre-GA group (404.74 ± 97.73 pre‐GA *vs* 679.26 ± 169.32 post‐GA, *P* < 0.001), whereas no differences were observed when comparing treatment with serum from the post-LA group to the pre-LA group (456.88 ± 146.78 pre‐LA *vs* 506.67 ± 125.69 post‐LA, *P* = 0.40). Furthermore, there was a statistically significant difference between the GA and LA groups when comparing the mean percentage change from post- to preoperative values in cell invasion (68.09% ± 30.83% in the GA group *vs* 14.07% ± 16.88% in the LA group, *P <*0.001) (**Figure 2F**).

### Serum From RFA Patients Receiving GA Facilitated Cell Migration of HepG2 Cells

**Figure 3:**
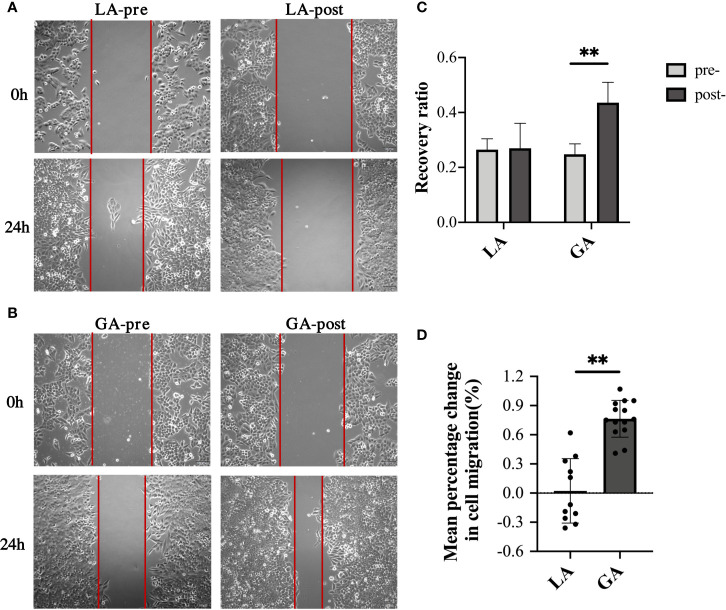
Serum from RFA patients receiving GA facilitated cell migration of HepG2 cells. **(A)** Representative image of HepG2 cells cultured with preoperative or 1h postoperative serum from the LA group; **(B)** Representative image of HepG2 cells cultured with preoperative or 1h postoperative serum from the GA group; **(C)** Graphical representation of recovery ratios of the four groups in **(A, B, D)** Graphical representation of the mean percentage change from post- to preoperative values of recovery ratios in the GA group *vs* the LA group. Values are expressed as mean ± SD. *n* =11, LA group. *n* = 14, GA group. **P < 0.01. GA, General Anesthesia; LA, Local Anesthesia.

Scratch assays were performed to examine cell migration ability of HepG2 cells treated with the different serum. Results were consistent with their respective invasion abilities, showing that while serum from pre- and postoperative LA patients had similar effects on the migration ability of HepG2 cells, postoperative serum from GA patients significantly promoted HepG2 invasion compared to preoperative serum (0.26 ± 0.05 pre-LA *vs* 0.27 ± 0.09 post-LA, *P* = 0.86; 0.25 ± 0.04 pre‐GA *vs* 0.44 ± 0.07 post‐GA, *P <*0.001; 76.43% ± 18.96% change in the GA group *vs* 2.27% ± 33.17% change in the LA group, *P <*0.001) (**Figure 3**).

### Serum From RFA Patients Receiving GA Facilitated Cell Proliferation of HepG2 Cells After Long-Term, but Not Short-Term, Exposure

**Figure 4:**
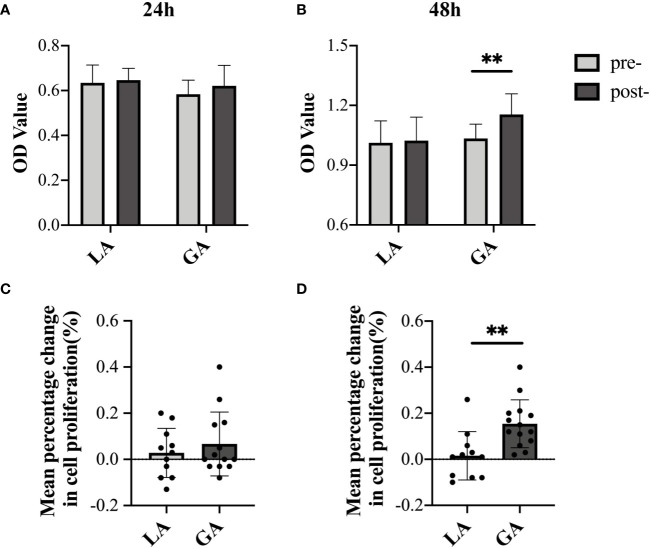
Serum from RFA patients receiving GA facilitated cell proliferation of HepG2 cells after long-term, but not short-term, exposure. **(A)** CCK-8 assay OD values (culture time = 24h); **(B)** CCK-8 assay OD values (culture time = 48h); **(C)** mean percentage change from post- to preoperative OD value (culture time = 24h); **(D)** mean percentage change from post- to preoperative OD value (culture time = 48h). Values are expressed as mean ± SD. *n* = 11, LA group. *n* = 13~14, GA group. **P < 0.01. GA, General Anesthesia; LA, Local Anesthesia.

Next, CCK-8 assays were used to investigate the proliferative effects on HepG2 cells when using serum from both groups. Cellular proliferation did not differ between any of the groups when cultured for 24h in post- *versus* preoperative serum (**Figures 4A, C**). To explore whether this was an exposure time issue, we extended the incubation time to 48h. Interestingly, as shown in **Figures 4B, D**, cells cultured in postoperative GA serum for 48h displayed a modest, but still significantly higher OD value than those in preoperative GA serum (1.03 ± 0.07 pre‐GA *vs* 1.15 ± 0.10 post‐GA, *P* = 0.001). There still was no observable difference in OD values in cells exposed to postoperative LA serum for 48h *versus* preoperative LA serum (1.01 ± 0.11 pre-LA *vs* 1.02 ± 0.12 post-LA, *P* = 0.81). The mean percentage change from post- to preoperative values in cell proliferation after 48h of culture was also significantly increased in the GA group compared to the LA group (15.43% ± 10.40% in the GA group *vs* 1.55% ± 10.48% in the LA group, *P* = 0.003) (**Figure 4D**).

**Figure 5:**
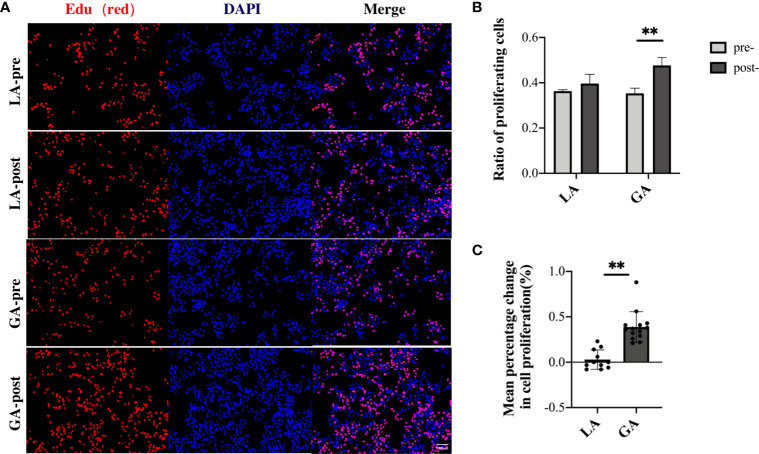
Serum from RFA patients receiving GA facilitated cell proliferation of HepG2 cells after long-term exposure, demonstrated with EdU assays. **(A)** HepG2 cells cultured with preoperative and 1h postoperative serum from the LA and GA groups, respectively; proliferative cells were stained with EdU (red), and nuclei were counterstained with Hoechst 33342 (blue); Original magnification, 100X; **(B)** Graphical representation of the proportions of EdU positive cells of the four groups; **(C)** Graphical representation of the mean percentage change from post- to preoperative values in EdU positive cells in the GA *vs* LA groups. Values are expressed as mean ± SD. *n* = 11, LA group. *n* = 14, GA group. **P < 0.01. GA, General Anesthesia; LA, Local Anesthesia.

In order to further verify these results, EdU assays were carried out to detect the proportion of cells involved in the proliferation phase after culturing for 48h in pre- and postoperative patient serum. The results were in agreement with the CCK-8 assay results above, showing that long-term exposure to post- GA serum, but not LA serum, caused a significant increase in proliferation activity of HepG2 cells (**Figures 5A–C**).

### Serum From RFA Patients Receiving GA Contained Increased Levels of Pro-Inflammatory Cytokines and Decreased Levels of Lymphokines

To understand whether GA leads to changes in the composition of certain molecules in patients’ serum, we then examined expression levels of several cytokines in patient serum. There was no difference in the levels of IL-1β, IL-6, TNF-α, IFN-γ, and IL-2 in preoperative serum between the two groups. In patients undergoing GA, serum pro-inflammatory cytokines, including IL-1β, IL-6 and TNF-α, increased significantly after surgery, while in the post- LA group, serum expression levels of these cytokines remained similar compared to the preoperative values from the same group. In addition, lymphokines including IFN-γ and IL-2 were significantly decreased after surgery in the GA group (**Table 2**).

**Table 2:** Levels of pro-inflammatory cytokines and lymphokines of the thermal ablation surgery patients [Mean (SD)].

Cytokines	LA (*n* = 8)		*P* value	GA (*n* = 8)		*P* value
	Pre-	Post-		Pre-	Post-	
IL-1β (ng/ml)	0.45 (0.29)	0.50 (0.36)	0.73	0.62 (0.22)	2.69 (2.22)	0.02
IL-6 (ng/ml)	1.19 (0.86)	3.52 (4.10)	0.36	3.25 (1.90)	26.48 (17.82)	0.00
lg (TNF-α) (ng/ml)	1.18 (0.70)	1.18 (0.61)	0.99	1.33 (0.50)	2.49 (0.64)	0.00
IFN-γ (ng/ml)	27.69 (3.52)	29.45 (3.19)	0.31	26.73 (6.43)	20.97 (3.48)	0.04
IL-2 (ng/ml)	24.77 (1.58)	25.34 (1.84)	0.52	25.26 (1.12)	23.52 (0.92)	0.01

Variables are shown as “mean (SD)”. IL-1β, interleukin-1β; IL-6, interleukin-6; TNF-α, Tumor necrosis factor-α; IFN-γ, interferon-γ; IL-2, interleukin-2; SD, standard deviation; LA, local anesthesia; GA, general anesthesia.

## Discussion

In the present study, we conducted a randomized and controlled study using serum from patients with HCC undergoing RFA, who received either GA or LA. We found that postoperative serum from patients who received GA, but not those who received LA, were able to significantly promote the invasion, migration and proliferation ability of a human HCC cell line. HepG2 cells also displayed upregulated expression levels of pro-inflammatory cytokines and downregulated levels of lymphokines when treated with post- GA serum. Although a direct and definite causal relationship between anesthesia method and tumor-promoting features in serum remains to be verified, these findings suggest that GA is probably associated with a poorer prognosis for HCC patients receiving RFA surgery compared to patients who received only LA.

Whether anesthetic drugs and anesthesia methods influence the prognosis of cancer patients has been a topic of interest in recent years. Several retrospective clinical studies have shown that cancer patients receiving GA combined with regional anesthesia have a better prognosis than patients undergoing surgery under GA alone (10), whereas several large clinical studies published in recent years, both prospectively and retrospectively, show that anesthesia methods have no effect on patients’ OS or RFS (16–18). Meanwhile, most fundamental studies focusing on anesthetics have proven that propofol, midazolam and local anesthetics exert potential anti-cancer properties, and in contrast, inhalants and opioids promote cancer development (19–24), which may be related to inhibition of the body’s immune function and upregulation of tumor cell proliferation. While the exact effects of anesthesia remain to be elucidated, it is important to note that most prior clinical studies were comparisons between GA and GA combined with regional anesthesia [epidural anesthesia or peripheral nerve block]. Therefore, the differences found in their clinical patients could possibly be due to a negative impact of GA, or a protective effect of regional anesthesia, or a combination of both could possibly lead to no differences between groups. Therefore, in the present study, patients undergoing RFA surgery for HCC are included and studied, since RFA is a unique tumor surgery that can verify the association between GA *per se* and tumor malignancy.

According to our previous multi-center retrospective cohort study, HCC patients who received RFA surgery under GA have a higher rate of tumor recurrence and shorter OS than those who received LA, however, the mechanism behind this phenomenon still remains unclear. In this randomized clinical trial, we found that patients’ serum from the post-GA group significantly promoted the invasion and migration ability of HepG2. The ability of cancer cells to migrate and invade directly relates to their degree of malignancy during cancer development (25). Increased invasion and migration abilities of cancer cells allow them to change position within tissues more easily, and once they arrive at suitable sites, such as bone and lung, metastasis occurs (26). Therefore, our findings that HepG2 cells display higher abilities of invasion and migration in serum from postoperative GA patients compared to preoperative patients, indicates that GA may promote remote metastasis of HCC, resulting in a poorer prognosis. In addition, long-term (48h), but not short-term (24h), exposure to postoperative serum from patients of the GA group caused a significant increase in proliferation activity of HepG2 cells. We speculated that this delayed increase is due to a much lower biological activity of human serum compared to fetal calf serum, which is normally used in cell culture experiments. An extended time frame is probably needed for HepG2 cells to reach the logarithmic phase when using adult patient serum.

The body’s immune system plays an important role in resisting tumor recurrence and metastasis. Many studies have shown that the function of multiple immune cells, including natural killer cells, effector T cells, lymphocytes, dendritic cells and B cells, are suppressed after GA (27, 28). Lymphokines are a kind of cytokine derived from lymphocytes, which suppress tumor progression and metastasis (29–31). IFN-γ, one of the major lymphokines, acts as an important immune-activated factor in cancer (31). Another lymphokine, IL-2, also plays a vital role in promoting the secretion of T cell cytokines, enhancing the killing ability of Natural Killer (NK) cells, and promoting B cells to participate in humoral immunity. In recent years, many studies have confirmed that the enhanced function of IL-2 can inhibit tumor occurrence and development (30). Our study showed that post- GA, levels of IL-2 and IFN-γ in patients’ serum decreased compared to pre- GA, suggesting that GA may lead to immunosuppression in patients through the inhibition of lymphocytes. This may arise from various anesthetics used during GA, especially opioids (32).

In contrast to lymphocytes, pro-inflammatory cytokines are associated with enhanced tumor development and spread (33). In this study, serum levels of pro‐inflammatory cytokines IL-1β, IL-6, TNF-α were significantly increased after GA, whereas in the LA group, levels of these cytokines only increased slightly in postoperative serum compared to preoperative serum. We speculate that this slight increase in the LA group reflects an increased inflammatory response caused by surgical trauma (34), and the significant increase in the GA group was due to a combination of both surgery trauma and GA. In addition to pro-inflammatory cytokines, it has been reported that many anesthetics used during GA also increase synthesis of the vascular endothelial growth factor, hypoxia-inducible factor and matrix metalloproteinase, which ultimately stimulate the proliferation and migration capacities of tumor cells and increase stromal angiogenesis (20, 28, 35).

At present, whether the differences between the GA and LA groups were caused by the anesthesia technique, in other words, the state of GA *per se*, or by the anesthetics used, remains unknown. We speculate that the latter contributes more to the differences than the former. The most obvious change induced by the GA technique is a loss of state of consciousness. In an awake patient, the hypothalamic-pituitary-adrenal axis and the sympathetic nervous system, whose activation is widely accepted to be associated with immune suppression (36, 37), should be more highly activated than in an anesthetized patient. Therefore, from this point of view, GA should have resulted in a better prognosis for cancer patients compared to patients receiving LA, which seems contradictory to the current findings. However, there are many other physiological changes during GA, which may contribute to tumor growth. Although unclear yet, their roles cannot be simply ruled out currently. On the other hand, various anesthetics have been reported to influence environmental signals that affect tumor outcome (38–40). For example, opioids, which are widely utilized in perioperative clinical practice for analgesia, could, after binding to their receptors (i.e., μ-opioid receptor), activate Akt and mTOR signaling, a well-defined pathway that contributes to tumor survival (38). Even though several studies have shown that propofol has potential anti-cancer properties (39), a recent study by Liu et al. (40) demonstrates that propofol augments lung tumor metastasis by downregulating TRIM21 expression and consequently promoting adhesion and extension of tumor cells. Therefore, we assume that mixed influences from multiple anesthetics accounted for at least part of the differences that were observed in the current study between the two groups. Whether one or several medications among them played a major role remains to be elucidated by more studies.

This study has certain limitations. First, the sample size was relatively small. However, when taking into consideration the actual sample size of 25 and an observed difference of 54% in the primary outcome between the two groups, the actual calculated statistical power is much higher than the estimated power. Second, the objectives of this study were indirect indicators of tumor outcome. Follow-up studies of direct indicators, such as long-term RFS or OS, would provide convincing evidence whether anesthesia methods influence the prognosis of HCC patients.

In summary, these findings suggest that GA may affect the serum milieu of patients with HCC, thereby promoting the malignant biological behavior of HCC. These results provide important guidance for anesthesia method choice in HCC patients undergoing RFA surgery, and also indicate a necessity for large‐scale, multicenter, and prospective clinical studies in such patients, to further verify the influence of anesthesia methods on their long-term prognosis.

## Data Availability Statement

The original contributions presented in the study are included in the article/supplementary material. Further inquiries can be directed to the corresponding authors.

## Ethics Statement

The studies involving human participants were reviewed and approved by Institutional Human Ethics Committee of Renji Hospital. The patients/participants provided their written informed consent to participate in this study.

## Author Contributions

YS, TWu, and TWa have contributed equally to this work and share first authorship. BZ and JT were both corresponding authors. All authors contributed to the article and approved the submitted version.

## Funding

This study received financial support from the Shanghai Science and Technology Committee Foundation (grant number 19ZR1430600), Shanghai Municipal Health Commission Foundation (grant number ZY (2018-2020)-ZYJS-48), Clinical Research Plan of SHDC (grant number SHDC2020CR4062), and Shanghai Municipal Commission of Health and Family Planning (grant number 201840241).

## Conflict of Interest

The authors declare that the research was conducted in the absence of any commercial or financial relationships that could be construed as a potential conflict of interest.

## Publisher’s Note

All claims expressed in this article are solely those of the authors and do not necessarily represent those of their affiliated organizations, or those of the publisher, the editors and the reviewers. Any product that may be evaluated in this article, or claim that may be made by its manufacturer, is not guaranteed or endorsed by the publisher.
